# The usefulness of MMP-9, TIMP-1 and MMP-9/TIMP-1 ratio for diagnosis and assessment of COPD severity

**DOI:** 10.1186/s40001-023-01094-7

**Published:** 2023-03-20

**Authors:** Sanja Dimic-Janjic, Mir Alireza Hoda, Branislava Milenkovic, Jelena Kotur-Stevuljevic, Mihailo Stjepanovic, Daniela Gompelmann, Jelena Jankovic, Milica Miljkovic, Jelena Milin-Lazovic, Natasa Djurdjevic, Dragana Maric, Ivan Milivojevic, Spasoje Popevic

**Affiliations:** 1 Faculty of Medicine, University of Belgrade, Clinic for Pulmonology, University Clinical Center of Serbia, Dr Subotica 8, Belgrade, Serbia; 2 Clinic for Pulmonology, University Clinical Center of Serbia, Koste Todorovica 26, Belgrade, Serbia; 3 Department of Thoracic Surgery, Medical University of Vienna, Vienna, Austria; 4 Faculty of Pharmacy, Department for Medical Biochemistry, University of Belgrade, Belgrade, Serbia; 5 Division of Pulmonology, Department of Internal Medicine II, Medical University of Vienna, Vienna, Austria; 6 Faculty of Medicine, Institute for Medical Statistics and Informatics, University of Belgrade, Belgrade, Serbia

**Keywords:** Chronic obstructive pulmonary disease, Matrix metalloproteinase-9, Tissue inhibitor of metalloproteinase-1, Biomarkers, Airflow limitation, Lung hyperinflation

## Abstract

**Background:**

Inflammation, oxidative stress and an imbalance between proteases and protease inhibitors are recognized pathophysiological features of chronic obstructive pulmonary disease (COPD). The aim of this study was to evaluate serum levels of matrix metalloproteinase-9 (MMP-9) and tissue inhibitor of metalloproteinase-1 (TIMP-1) in patients with COPD and to assess their relationship with lung function, symptom severity scores and recent acute exacerbations.

**Methods:**

In this observational cohort study, serum levels of MMP-9 and TIMP-1 and the MMP-9/TIMP-1 ratio in the peripheral blood of COPD patients with stable disease and healthy controls were determined, and their association with lung function (postbronchodilator spirometry, body plethysmography, single breath diffusion capacity for carbon monoxide), symptom severity scores (mMRC and CAT) and exacerbation history were assessed.

**Results:**

COPD patients (*n* = 98) had significantly higher levels of serum MMP-9 and TIMP-1 and a higher MMP-9/TIMP-1 ratio than healthy controls (*n* = 47) (*p* ≤ 0.001). The areas under the receiver operating characteristic curve for MMP-9, TIMP-1 and the MMP-9/TIMP-1 ratio for COPD diagnosis were 0.974, 0.961 and 0.910, respectively (all *p* < 0.05). MMP-9 and the MMP-9/TIMP-1 ratio were both negatively correlated with FVC, FEV_1,_ FEV_1_/FVC, VC, and IC (all *p* < 0.05). For MMP-9, a positive correlation was found with RV/TLC% (*p* = 0.005), and a positive correlation was found for the MMP-9/TIMP-1 ratio with RV% and RV/TLC% (*p* = 0.013 and 0.002, respectively). Patients with COPD GOLD 3 and 4 presented greater MMP-9 levels and a greater MMP-9/TIMP-1 ratio compared to GOLD 1 and 2 patients (*p* ≤ 0.001). No correlation between diffusion capacity for carbon monoxide and number of acute exacerbations in the previous year was found.

**Conclusions:**

COPD patients have elevated serum levels of MMP-9 and TIMP-1 and MMP-9/TIMP-1 ratio. COPD patients have an imbalance between MMP-9 and TIMP-1 in favor of a pro-proteolytic environment, which overall indicates the importance of the MMP-9/TIMP-1 ratio as a potential biomarker for COPD diagnosis and severity.

## Background

Chronic obstructive pulmonary disease (COPD) is a heterogeneous disorder characterized by airflow limitation associated with an abnormal inflammatory response in the lungs [1]. Inflammation and oxidative stress can affect protease homeostasis, causing enhanced extracellular matrix (ECM) degradation and leading to progressive alveolar destruction and airway remodeling [2]. Matrix metalloproteases (MMPs) have been implicated as one of the many inflammatory mediators involved in COPD pathogenesis. MMPs degrade the ECM and have a complex relationship with cytokines, chemokines, adhesion receptors, growth factors and their receptors and a variety of enzymes [3–5]. Matrix metalloproteinase-9 (MMP-9) is a type IV collagen-degrading MMP secreted in the lungs by neutrophils, alveolar macrophages, bronchial epithelial cells and eosinophils [6, 7]. MMP-9 plays a significant role in augmenting the inflammatory process, matrix turnover and lung repair [8]. Its activity is regulated in three ways: transcription, proteolytic activation of the zymogen form and inhibition of the active enzyme by a host of natural inhibitors [9–11]. Inflammation and lung tissue destruction are intensified by the synergistic action of proteinases and reactive oxygen species [12]. It is well documented that pro-oxidants can activate the proenzyme form of MMPs and subsequently inactivate MMPs to reach homeostatic balance in affected tissue [13]. Tissue inhibitor of metalloproteinase (TIMP-1) is an inhibitor of MMP-9 that binds to MMP-9 precursors and to the active form. Deregulation and imbalance between the activity of proteases and protease inhibitors has implications for COPD pathogenesis.

Expiratory flow limitation (EFL) in COPD arises due to emphysema and airway disorder [14]. The current diagnosis and classification of COPD relies significantly on the degree of EFL derived by spirometric forced expiratory volume in 1 second (FEV_1_) measurement. Spirometry is the gold standard for accurate and reproducible measurements of lung function [15]. Lung hyperinflation is an important clinical phenomenon and is related to expiratory airflow limitation. Body plethysmography remains a reliable gold standard for the measurement of lung volumes and other compartments related to lung hyperinflation [16]. Diffusing capacity for carbon monoxide (DLCO) is one of the established physiological measurements of emphysema severity [17].

The aim of this study was to evaluate serum levels of both MMP-9 and TIMP-1 in COPD patients and to assess their relationship with lung function, symptom severity scores and acute exacerbations of chronic obstructive pulmonary disease (AECOPD). We hypothesized that elevated MMP-9 and an increased MMP-9/TIMP-1 ratio would be identified in patients with the most pronounced lung function impairment, increased burden of symptoms and more frequent AECOPD.

## Material and methods

### Study population

In this prospective study, COPD patients and healthy controls who were referred to the hospital-based Outpatient Pulmonology Department in the University Clinical Center of Serbia, Belgrade, between May 2017 and January 2018 were enrolled.

### Subject enrollment and assessment of MMP-9 and TIMP-1

In this observational cohort study, 98 COPD patients and 47 healthy subjects were enrolled.

Inclusion and exclusion criteria for COPD patients are summarized in Table 1. All COPD patients’ inhalation therapy and exacerbation history in the previous year were recorded. Symptoms were assessed by the Modified Medical Research Council Dyspnea Scale (mMRC) and COPD Assessment Test (CAT), translated and validated [18]. COPD exacerbation was defined as acute worsening of respiratory symptoms that results in the requirement of additional therapy. A frequent exacerbator was a COPD patient with ≥ 2 exacerbations per year [19].

**Table 1:** Inclusion and exclusion criteria

Inclusion criteria	Exclusion criteria
Adults ≥ 40 years of age	Diagnosis of asthma or any other chronic respiratory disease
Diagnosis of COPD at least 6 months prior to enrollment	Immunosuppression of any kind
Diagnosis according to the Global Initiative for Chronic Obstructive Disease (GOLD)	Refusal to give informed consent
At least one spirometry measurement in the last year with a postbronchodilator, FEV_1_/FVC < 0.70	Any acute or chronic condition that would limit the ability of the patient to participate in the study
Stable disease (no recent exacerbation for at least 4 weeks prior to enrollment)	

The control group included 47 healthy adults ≥ 40 years of age with no pulmonary, cardiovascular or other chronic disease, infection or inflammation that could influence inflammatory and oxidative status.

In all subjects (COPD patients and healthy controls), age, sex, smoking status (defined as current/former/never and reported in pack/years), and self-reported comorbidities were recorded. A current smoker was defined as someone who either smokes every day (daily smoker) or who currently smokes but not every day (occasional or nondaily smoker) [20]. Former smoker referred to someone who has smoked more than 100 cigarettes in their lifetime but has not smoked in the last 12 months.

All subjects underwent blood sampling and determination of serum MMP-9 and TIMP-1 levels with a minimum time frame of 4 weeks after previous AECOPD. Blood samples were collected and evaluated for complete blood count (CBS) and white blood cell count (WBC) using an LH750^®^ Hematology Analyzer (Beckman Coulter, Inc., Brea, California, USA) and for serum separation. After collection of whole blood using Vacutainer tubes, samples were stored undisturbed at room temperature for 30 min and then centrifuged at 1000 × g for 10 min. Serum samples were aliquoted into 0.5 mL portions and stored at − 80 °C until analysis. MMP-9 and TIMP-1 were measured using enzyme-linked immunosorbent assay (ELISA) kits according to the manufacturer’s instructions (Quantikine ELISA, R&D Systems, Minneapolis, USA).

### Pulmonary function tests

Pulmonary function tests were performed according to American Thoracic Society/European Respiratory Society (ATS/ERS) criteria [21–24]. Postbronchodilator spirometry was recorded on a JAEGER^®^ MasterScreen Pneumo, single breath diffusion capacity for carbon monoxide (CO) on a JAEGER^®^ MasterScreen Diffusion and body plethysmography on a JAEGER^®^ MasterScreen Body. The analyzed parameters were forced expiratory volume in 1 second (FEV1), forced vital capacity (FVC), the FEV1/FVC ratio, diffusion capacity for carbon monoxide (DLCO), carbon monoxide transfer coefficient (KCO), total lung capacity (TLC), residual volume (RV), vital capacity (VC), inspiratory capacity (IC), expiratory reserve volume (ERV) and total resistance (R tot).

### Statistical analysis

Descriptive statistics, including measures of central tendency (arithmetic mean, median and mode), measures of variability (range, interquartile range and standard deviation) and relative numbers (structure indicators), were calculated for all study variables of interest. Graphical and mathematical procedures were used to test the normality of distribution. To test the statistical hypothesis, in accordance with the type and distribution of variables, the *t* test, Mann‒Whitney *U* test, chi-square test and Fisher’s test were used. To assess the correlation between variables, Pearson’s correlation analyses or Spearman rank correlation were performed. A receiver operating characteristic (ROC) curve was used to determine cutoff values for continuous variables. To identify potential predictors of COPD, univariate and multivariate logistic regression analyses were performed. All statistical tests were executed using a significance level of *P* < 0.05. Analyses were performed in IBM SPSS Statistics for Windows, version 26.0 (Armonk, NY: IBM Corp., 2019). MedCalc for Windows, version 19.4 (MedCalc Software, Ostend, Belgium), was used for figure presentation.

## Results

### Clinical characteristics and comparison between COPD patients and healthy subjects

In this observational cohort study, 98 COPD patients (mean age 63.4 years [95% CI 62.1–65.8], male 58.2%, current smokers 35.7% [median 40 (10–100) pack/years), former smokers 64.3% [median 30 (5–100) pack/years]) were enrolled and compared to 47 healthy subjects (mean 61.7 years [95% CI 60.5–63 years], male 46.8%, current smokers 29.8%).

There were no significant differences in age, sex or smoking status between the two study groups. Serum MMP-9 and TIMP-1 levels and the MMP-9/TIMP-1 ratio were significantly higher in COPD patients than in healthy controls (*p* ≤ 0.001) (Table 2).

**Table 2:** Demographics, MMP-9 and TIMP-1 levels and the MMP-9/TIMP-1 ratio in COPD patients and healthy controls

	COPD (*n* = 98)	Healthy controls (*n* = 47)	*p*
Age, years, mean ± SD^a^	63.4 ± 4.3	61.7 ± 4.3	0.123
Male, *n* (%)^b^	58.2%	46.8%	
Female, *n* (%)	41.8%	53.2%	0.199
Current smokers, *n* (%)^b^	35 (35.7%)	14 (29.8%)	0.489
MMP-9 (ng/ml), med (95% CI)^c^	848.7 (728.6–981.5)	58.7 (47.9–76.2)	p ≤ 0.001
TIMP 1 (ng/ml), med (95% CI)^c^	234.7 (222.5–246.1)	82.3 (73.0–89.7)	p ≤ 0.001
MMP-9/TIMP-1 ratio, med (95% CI)^c^	3.4 (2.9–4.2)	0.40 (0.3–0.5)	p ≤ 0.001

*SD* standard deviation, *MMP-9* matrix metalloproteinase 9, *TIMP-1* tissue inhibitor of metalloproteinase 1

^a^Data were compared by independent *T* test

^b^Data were compared by chi-square test

^c^Data were compared by Mann‒Whitney test

### Sensitivity and specificity of MMP-9 and TIMP-1 for COPD diagnosis

**Fig. 1:**
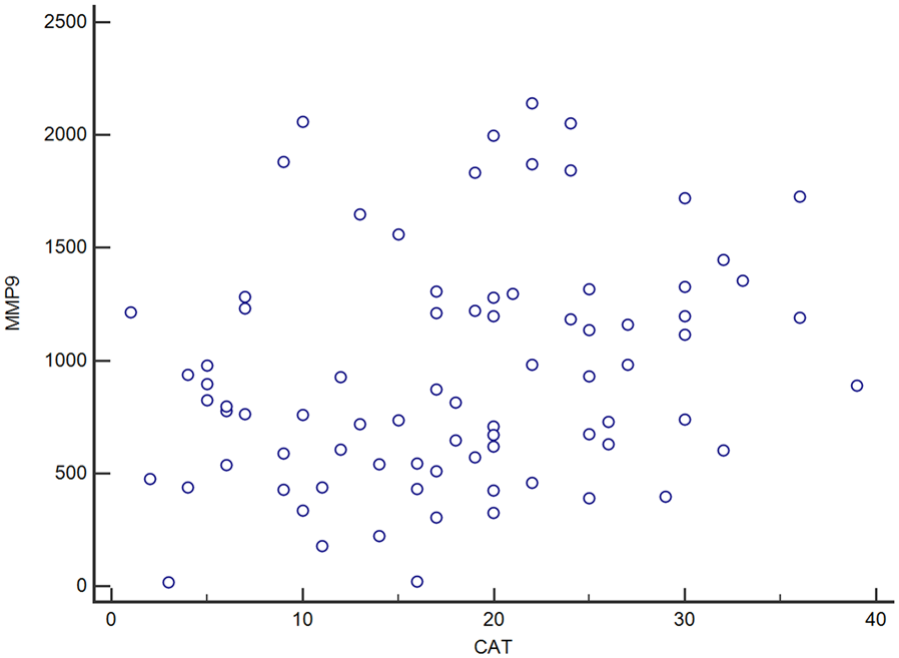
ROC curve for MMP-9/TIMP-1 ratio for COPD diagnosis

The ROC curves for MMP-9, TIMP-1 and the MMP-9/TIMP-1 ratio for COPD diagnosis are presented in Table 3, with areas under the curve (AUCs) of 0.974, 0.961 and 0.910, respectively. An MMP-9 value of 612.8 ng/ml or greater was 70.0% sensitive and 100% specific for COPD diagnosis. TIMP-1 equal to or greater than 110.7 ng/ml was 100% sensitive and 81% specific for COPD diagnosis. An MMP-9/TIMP-1 ratio value of 1.16 or greater was 96% sensitive and 91% specific for COPD diagnosis (Fig. 1).

**Table 3:** Areas under the curve (AUCs) for MMP-9, TIMP-1 and the MMP-9/TIMP-1 ratio for COPD diagnosis

	AUC	*p*	95% CI	UL
			LL	
MMP-9 (ng/ml)	0.974	*p* ≤ 0.001	0.941	1.000
TIMP-1(ng/ml)	0.961	0.022	0.918	1.000
MMP-9/TIMP-1 ratio (ng/ml)	0.91	*p* ≤ 0.001	0.836	0.984

*AUC* area under the curve, *LL* lower limit, *UL* upper limit

### Association of MMP-9, TIMP-1 and MMP-9/TIMP-1 ratio with lung function, disease severity, COPD symptoms and exacerbation history

#### Functional and clinical characteristics of the COPD patients

The functional and clinical characteristics of the COPD patients are presented in Table 4. Of all COPD patients, 65.3% had comorbidities, and 50% had cardiovascular comorbidities. COPD patients had severe airflow limitation, impaired diffusion capacity, lung hyperinflation and elevated symptom scores (Table 4).

**Table 4:** Clinical parameters in COPD patients

	*n* = 98	95% CI	
		lower CI	upper CI
AECOPD/previous year, med (IQR)	2 (2)	2.0	3.0
COPD duration in years, med (IQR)	7 (6)	6.0	8.0
CAT score, med (IQR)	18.5 (14.2)	16.0	20.0
mMRC score, med (IQR)	2.0 (2.0)	2.0	3.0
CRP, med (IQR)	3.4 (6.2)	2.4	4.6
WBC (10 ^ 9/l), mean ± SD	8.4 ± 2.6	7.9	8.9
Serum neutrophils (10 ^ 9/l), mean ± SD	5.4 ± 2.3	5.0	5.9
Serum eosinophils (10 ^ 9/l), mean ± SD	0.2 ± 0.2	0.2	0.2
Serum eosinophils, (%)	2.3 ± 1.7	2.0	2.7
Serum lymphocytes (10 ^ 9/l), mean ± SD	2.1 ± 0.8	1.9	2.3
FEV_1/_FVC, mean ± SD	43.7 ± 12.6	41.2	46.3
Post-BD FVC% predicted, mean ± SD	85.6 ± 19.4	81.7	89.5
Post-BD FEV_1_% predicted, mean ± SD	47 ± 18.6	43.3	50.8
DLCO% predicted, mean ± SD	50.1 ± 21.8	45.8	54.5
KCO% predicted, mean ± SD	60.8 ± 24.1	55.8	65.7
TLC% predicted, mean ± SD	125.5 ± 23.5	120.7	130.3
RV% predicted, mean ± SD	194.6 ± 63.4	181.7	207.6
VC% predicted, mean ± SD	91.3 ± 19.5	87.3	95.3
RV% TLC predicted, mean ± SD	146.4 ± 28.1	140.7	152.1
R tot, mean ± SD	0.6 ± 0.3	0.5	0.6
IC% predicted, mean ± SD	85.1 ± 30	79.0	91.3
ERV% predicted, mean ± SD	112.0 (66.0)	97.9	121.4

Data are expressed as the mean ± SD, *n* (%) or med (95% CI)

*BD* bronchodilator, *AECOPD* acute exacerbation of chronic obstructive disease, *FEV1* forced expiratory volume in 1 s, *FVC* forced vital capacity, *DLCO* diffusion capacity for carbon monoxide (CO), *KCO* carbon monoxide transfer coefficient, *TLC* total lung capacity, *RV* residual volume, *VC* vital capacity, *IC* inspiratory capacity, *ERV* expiratory reserve volume, *R tot* total resistance, *WBC count* white blood cell count, *CRP* C-reactive protein, *CAT* COPD Assessment Test, *MMP-9* matrix metalloproteinase 9, *TIMP-1* tissue inhibitor of metalloproteinase 1

#### Association of MMP-9, TIMP-1 and the MMP-9/TIMP-1 ratio with lung function, COPD symptoms and CBC

Negative correlations were identified between MMP-9, the MMP-9/TIMP-1 ratio and the following post-BD spirometry and body plethysmography parameters: FVC, FEV_1,_ FEV_1_/FVC, VC and IC (Table 5). Positive correlations were identified between both MMP-9 and the MMP-9/TIMP-1 ratio with RV and RV% TLC (Table 5). No correlation was found between serum TIMP-1 and any lung function parameter.

**Table 5:** Correlation of MMP-9, TIMP-1, and MMP-9/TIMP-1 ratio with lung function, clinical and CBC parameters

	MMP-9		TIMP-1		MMP-9/TIMP-1	
	*r*	*p*	*r*	*p*	*r*	*p*
FEV_1_/FVC	− 0.308	0.005	− 0.085	0.456	− 0.330	0.003
FVC (%) pred	− 0.294	0.008	− 0.043	0.704	− 0.282	0.011
FEV_1_ (%) pred	− 0.367	*p* ≤ 0.001	− 0.054	0.636	− 0.392	*p* ≤ 0.001
DLCO (%) pred	− 0.154	0.172	− 0.117	0.302	− 0.153	0.175
TLC (%) pred	0.012	0.918	− 0.119	0.293	0.105	0.355
RV (%) pred	0.220	0.050	− 0.031	0.786	0.277	0.013
VC (%) pred	− 0.323	0.003	− 0.152	0.178	− 0.263	0.018
RV% TLC pred	0.308	0.005	0.015	0.895	0.335	0.002
IC (%) pred	− 0.359	*p* ≤ 0.001	− 0.148	0.189	− 0.291	0.009
BMI (kg/m^2^)	0.157	0.164	0.289	0.009	0.043	0.703
CRP (mg/l)	0.301	0.007	0.370	0.001	0.164	0.148
WBC (10 ^ 9/l)	0.462	*p* ≤ 0.001	0.293	0.009	0.339	0.002
Ne (10 ^ 9/l)	0.478	*p* ≤ 0.001	0.346	0.002	0.322	0.004
Ne (%)	0.315	0.005	0.225	0.046	0.194	0.086
Eo (10 ^ 9/l)	0.016	0.890	0.007	0.949	0.045	0.698
Eo (%)	− 0.140	0.222	− 0.175	0.125	−0.038	0.743
Hgb (g/l)	0.352	0.002	0.263	0.020	0.243	0.032
CAT	0.273	0.014	0.053	0.641	0.312	0.005
mMRC	0.288	0.010	0.103	0.365	0.281	0.011

*FEV*_*1*_ forced expiratory volume in 1 s, *FVC* forced vital capacity, *DLCO* diffusion capacity for carbon monoxide (CO), *KCO* carbon monoxide transfer coefficient, *TLC* total lung capacity, *RV* residual volume, *VC* vital capacity, *IC* inspiratory capacity, *ERV* expiratory reserve volume, *R tot* total resistance, *WBC count* white blood cell count, *CRP* C- reactive protein, *Ne* absolute neutrophil count, *Hgb* hemoglobin, *CAT* COPD Assessment Test, *mMRC* modified Medical Research Council, *MMP-9* matrix metalloproteinase 9, *TIMP-1* tissue inhibitor of metalloproteinase 1

**Fig. 2:**
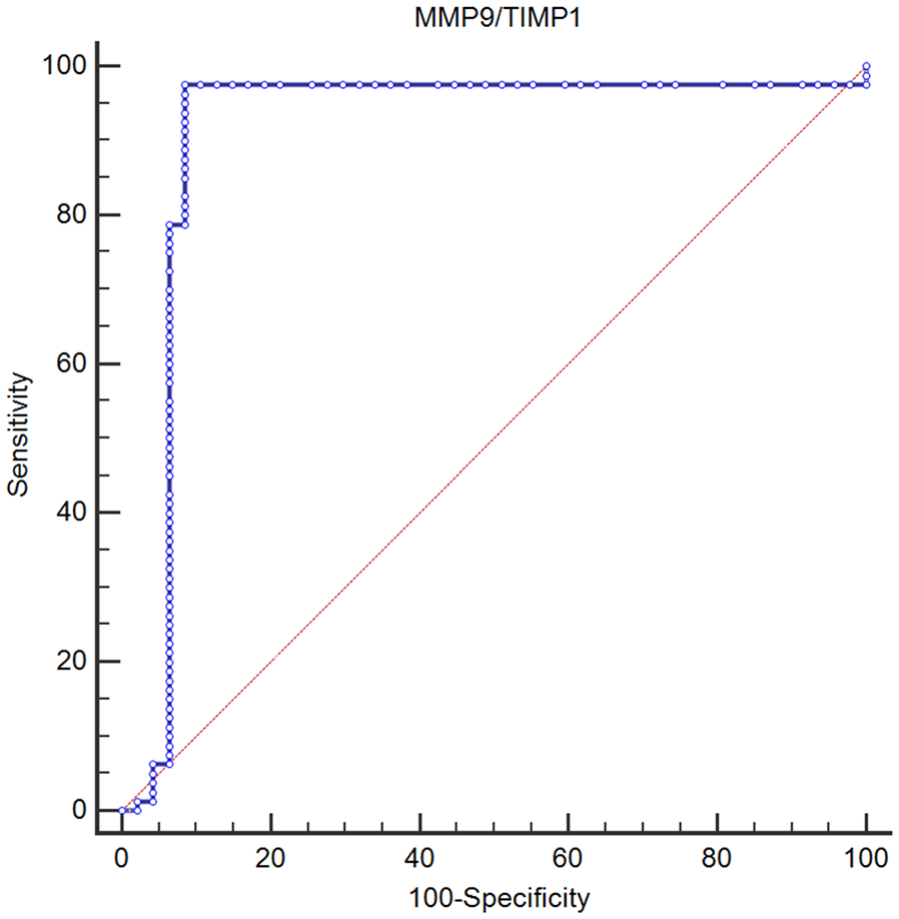
Correlation of MMP-9 with CAT

No statistically significant correlation between MMP-9, TIMP-1, the MMP-9/TIMP-1 ratio and diffusion capacity for carbon monoxide (DLCO) or transfer coefficient for carbon monoxide (KCO) was found (Table 5). CAT and mMRC scores were positively associated with MMP-9 and the MMP-9/TIMP-1 ratio (Fig. 2). MMP-9 and TIMP-1 were positively associated with CRP. MMP-9, TIMP-1 and the MMP-9/TIMP-1 ratio were positively associated with leukocytes, absolute neutrophil count and hemoglobin levels.

#### Association of MMP-9, TIMP-1 and the MMP-9/TIMP-1 ratio with disease severity and exacerbation history

Both MMP-9 and the MMP-9/TIMP-1 ratio were statistically greater in COPD patients in GOLD stages 3 and 4 (*p* = 0.003; *p* ≤ 0.001) and were also higher in the patients with AECOPD ≥ 2 but failed to reach statistical significance (Table 6).

**Table 6:** MMP-9, TIMP-1, and the MMP-9/TIMP-1 ratio according to GOLD stages and number of AECOPD (events/year)

	GOLD 1 and 2 *N* = 40	GOLD 3 and 4 *N* = 58	*p*	Exacerbations < 2 *N* = 41	Exacerbations ≥ 2 *N* = 57	*p*
MMP-9 (ng/ml), med (95% CI)^b^	596.8 (437.9–761.5)	980 (825.5–1195.6)	0.003	778.5 (540.6–981.5)	892.3 (728.6–1188.9)	0.196
TIMP-1(ng/ml), med (95% CI)^b^	223.9 (208–245.2)	238.9 (228.9–249.3)	0.552	222.7 (198.9–253.9)	236.6 (228.9–248.4)	0.669
MMP-9/TIMP-1^b^ ratio (ng/ml), med (95% CI)	2.4 (2–3.2)	4.2 (3.3–5)	*p* ≤ 0.001	3.2 (2.4–4.8)	3.5 (3.1–4.6)	0.222

^a^Data were compared by independent *t* test

^b^Data were compared by Mann‒Whitney test

#### Association of MMP-9, TIMP-1 and the MMP-9/TIMP-1 ratio with inhalation therapy

Triple therapy consisting of a fixed-dose combination inhaler containing an inhaled corticosteroid and long-acting beta_2_ agonist (ICS/LABA) and a long-acting muscarinic antagonist inhaler (LAMA) was reported by 65 subjects (63.7%). Bronchodilator therapy was reported by 33 subjects (32.3%). Bronchodilators included LAMA monotherapy (11.8%), LABA monotherapy (0.9%), LAMA plus LABA inhalers (16.7%) and short-acting beta_2_ agonist (SABA) (2.9%). COPD patients receiving ICS/LABA and LAMA triple therapy had higher serum MMP-9 (958.7 ng/ml [95% CI 737.3–1198.6]) and a higher MMP-9/TIMP-1 ratio (3.8 [95% CI 3.1–5]) compared with COPD patients receiving bronchodilator therapy (MMP-9, 726.7 ng/ml [95% CI 473.7–889.4]; MMP-9/TIMP-1 ratio, 3 [95% CI 2.1–3.4]). The difference, however, neared statistical significance for MMP-9 (*P* = 0.063) and the MMP-9/TIMP-1 ratio (*p* = 0.098) for both treatment groups.

#### MMP-9/TIMP-1 as a predictor for COPD diagnosis and COPD severity

Logistic regression analysis for COPD diagnosis prediction (no COPD versus COPD) adjusted for age and sex identified the MMP-9/TIMP-1 ratio as a significant predictor for COPD presence (Table 7). When we analyzed predictors for COPD severity according to GOLD criteria (COPD GOLD I + II versus COPD GOLD III + IV), the MMP-9/TIMP-1 ratio was a significant predictor of severe COPD and remained significant after adjustment for age, sex and AECOPD (Table 8).

**Table 7:** MMP-9/TIMP-1 ratio as a predictor for COPD diagnosis

	B	OR	95% CI for OR		*p*
			Lower	Upper	
MMP-9/TIMP-1	0.664	1.943	1.481	2.551	< 0.001
Sex	− 0.341	0.711	0.3	1.681	0.437
Age (years)	0.051	1.053	0.987	1.123	0.118
Constant	− 3.678	0.025			0.092

**Table 8:** MMP-9/TIMP-1 as a predictor of COPD severity

	B	OR	95% CI for OR		*p*
			Lower	Upper	
Exacerbations ≥ 2	− 0.5	0.607	0.212	1.739	0.353
MMP-9/TIMP-1	0.375	1.456	1.086	1.951	0.012
Sex	0.139	1.150	0.415	3.183	0.788
Age (years)	0.018	1.018	0.958	1.081	0.566
Constant	− 1.732	0.177			0.46

## Discussion

Chronic exposure to tobacco smoke causes oxidative stress and airway inflammation, resulting in deregulation of protease activity, extracellular matrix degradation, progressive alveolar destruction and airway remodeling with significant functional impairment and irreversible loss of lung function. Airway remodeling includes structural changes caused by repeated injury and repair processes and longstanding airway inflammation [25]. Such deregulation is a hallmark of COPD pathogenesis [26].

Our study is the first to examine serum MMP-9, TIMP-1 and the MMP-9/TIMP-1 ratio for their predictive value for the presence of COPD and to assess the relationship with the full range of functional lung parameters obtained by spirometry, single breath diffusion capacity for CO, body plethysmography and clinical COPD indicators.

We found that serum values of MMP-9 and TIMP-1 and the MMP-9/TIMP-1 ratio were significantly higher in COPD patients than in healthy controls. This suggests protease/antiprotease deregulation and increased proteolytic activity in COPD patients. Similar results were presented in a meta-analysis published by Li and coworkers that included twenty relevant studies of a total of 923 COPD patients and 641 healthy controls (serum MMP-9 and TIMP-1 values were greater in COPD patients than in healthy controls) [27]. In the study by Gilowska and coauthors, COPD patients were predisposed to synthesize more MMP-9 and MMP-9/TIMP-1 complexes than healthy controls, most likely due to the diseased lung environment rather than to genetic features of the MMP-9 gene [28]. The study by Linder and coworkers found greater MMP-9 values in COPD patients and a significant correlation with productive cough and decreased FEV_1_, consistent with our findings [29]. Ilumets and coworkers demonstrated that MMP-9 levels did not change with age but were elevated in smokers and COPD patients compared to nonsmokers, providing a clear link to cigarette smoke-induced lung remodeling and COPD [30]. In a study by Piesiak and coworkers, serum MMP-9 and TIMP-1 levels were higher in COPD patients than in healthy controls, and both biomarkers correlated with CRP level and FEV_1_, which corresponds to the findings of our study [31]. However, serum MMP-9 levels and the MMP-9/TIMP-1 ratio seem to be associated with COPD. Several studies that investigated the potential role of MMP-9 in COPD demonstrated elevated MMP-9 in systemic and local compartments other than serum: sputum [32, 33], bronchoalveolar lavage fluid (BALF) [34] and plasma [35, 36]. The potential role of MMP-9 was assessed according to disease state, either stable or AECOPD. In a study by Wells and coworkers, an elevated plasma MMP-9 level was independently associated with AECOPD risk [37], and Stanojkovic and coworkers demonstrated that oxidant/antioxidant imbalance was significantly pronounced in patients with COPD exacerbation [38].

In the current study, we found the areas under the receiver operating characteristic curves for MMP-9, TIMP-1 and the MMP-9/TIMP-1 ratio for COPD diagnosis to be 0.974, 0.961 and 0.910, respectively. These results suggest that serum-based MMP-9 biomarkers add additional value to clinical characteristics predicting COPD.

Furthermore, we evaluated the association of MMP-9 and TIMP-1 with lung function parameters and found a negative correlation between serum MMP-9 level and the MMP-9/TIMP-1 ratio and the spirometric parameters FEV_1_/FVC ratio, FVC and FEV_1_. We failed to find a correlation between serum TIMP-1 and any lung function parameter. In a study by Ólafsdottir and coworkers, lower FEV_1_ values correlated with increased serum levels of MMP-9, MMP-9/TIMP-1 ratio and TIMP-1 [39]. The explanation could be related to the age of participants (888 participants over 70 years of age) and the fact that serum TIMP-1 level is age dependent. A negative correlation between MMP-9 and FEV_1_ has also been identified in the lung parenchyma in COPD patients [40] and airway secretions (sputum and BAL), as mentioned above. This suggests that serum MMP-9 and an increased MMP-9/TIMP 1 ratio are associated with airway dysfunction severity. In our study, MMP-9 increased significantly with the severity of airway obstruction, and TIMP-1 changed slightly, suggesting an imbalance between proteases and protease inhibitors culminating in enhanced proteolytic activity and indicating the importance of the MMP-9/TIMP-1 ratio as a potential biomarker in airway remodeling and COPD development.

Diffusion capacity for CO (DLCO) is considered a useful tool to assess emphysema severity. DLCO was significantly higher in COPD patients with lower GOLD stages (I and II) than in those with higher GOLD stages (III and IV). These results suggest that COPD patients with greater airway obstruction and higher GOLD stages had greater diffusion capacity impairment, indicating emphysema. However, we failed to substantiate our initial assumption that MMP-9, due to its role in ECM turnover, would correlate with DLCO as a functional measure of emphysema. Similarly, Beeh and colleagues demonstrated that both MMP-9 and TIMP-1 were elevated in COPD patient sputum, and MMP-9 negatively correlated with airway obstruction but not with either diffusion capacity or vital capacity [41]. Additionally, Papakonstantinou and colleagues demonstrated that MMPs and TIMPs failed to correlate with DLCO [42]. In the study by D’Armiento and coworkers, changes in MMP levels in plasma and bronchoalveolar lavage fluid correlated poorly with parameters of disease severity or progression, although they were greater in patients with emphysema [43]. Tsay and colleagues reported that serum MMP levels provided minimal additional information to improve the detection of mild emphysema [44]. Therefore, it is possible that the role of MMP-9 in the pathophysiology of COPD is more complex than previously considered and not only related to the development of emphysema.

In our study, circulating leukocytes and neutrophils positively correlated with serum MMP-9 and TIMP-1 levels and the MMP-9/TIMP-1 ratio and positively correlated with the number of AECOPD events. Sniteker and coworkers identified an increased correlation between MMP-9 and WBC count in smokers, supporting the theory that leukocytes are a potential source of circulating MMP-9 in vivo and that smoking affects the levels of both [45].

In the present study, triple therapy was the therapy of choice for patients with the most pronounced airway limitation and COPD symptoms. COPD patients on ICS/LABA + LAMA triple therapy had higher serum MMP-9 levels and a higher MMP-9/TIMP-1 ratio. In a study by Vlahos and coworkers, MMP-9 activity was elevated in severe COPD, closely related to neutrophilic inflammation, and in vivo, neutrophil activation was associated with glucocorticoid refractory release of MMP-9 [46] It is tempting to speculate that higher serum MMP-9 levels and a greater MMP-9/TIMP-1 ratio in patients with triple therapy is related to advanced disease and more exacerbations, but it is potentially an altered response to glucocorticoids.

COPD patients had a greater number of symptoms and more pronounced dyspnea as determined by mMRC and CAT. We found a significant association between MMP-9, the MMP-9/TIMP-1 ratio and mMRC and CAT scores but not with the number of recorded AECOPD cases in the previous year*.* Taking everything into consideration, it is possible that COPD patients with more stable phenotypes, who are not frequent exacerbators but experience increased dyspnea and symptom burden, exhibit greater proteolytic activity.

There are a few limitations to our study. First, ELISA was used to measure both total and pro-MMP-9. Therefore, it is possible that increased serum MMP-9 was not necessarily related to increased activity. Second, as blood samples were collected once, long-term elevation of serum MMP-9 cannot be substantiated. Third, the effects of serum MMP-9 and TIMP-1 reflect systemic features. We did not perform simultaneous analysis of MMP-9 and TIMP-1 in airway secretions as a reflection of the local compartment. The latter could potentially provide a more comprehensive assessment and should be the subject of future studies.

## Conclusion

COPD patients have elevated serum levels of MMP-9 and TIMP-1 and MMP-9/TIMP-1 ratio. COPD patients have an imbalance between MMP-9 and TIMP-1 in favor of a pro-proteolytic environment, which overall indicates the importance of the MMP-9/TIMP-1 ratio as a potential biomarker for COPD diagnosis and severity.

## Data Availability

The datasets used and/or analyzed during the current study are available from the corresponding author on reasonable request.

## Abbreviations

COPD: Chronic obstructive pulmonary disease
MMP-9: Matrix metalloproteinase-9
TIMP-1: Tissue inhibitor of metalloproteinase-1
ECM: Extracellular matrix
GOLD: Global Initiative for Chronic Obstructive Disease
ELISA: Enzyme-linked immunosorbent assay
EFL: Expiratory flow limitation
AECOPD: Acute exacerbation of chronic obstructive pulmonary disease
mMRC: Modified Medical Research Council Dyspnea Scale
CAT: COPD Assessment Test
FEV_1_: Forced expiratory volume in 1 second
FVC: Forced vital capacity
DLCO: Diffusion capacity for carbon monoxide
KCO: Carbon monoxide transfer coefficient
TLC: Total lung capacity
RV: Residual volume
VC: Vital capacity
IC: Inspiratory capacity
ERV: Expiratory reserve volume
R tot: Total resistance
CRP: C-reactive protein
LAMA: Long-acting muscarinic antagonist
IC: Inhaled corticosteroid
LABA: Long-acting beta_2_ agonist
BALF: Bronchoalveolar lavage fluid
